# Infection of the brown alga *E* *ctocarpus siliculosus* by the oomycete *E* *urychasma dicksonii* induces oxidative stress and halogen metabolism

**DOI:** 10.1111/pce.12533

**Published:** 2015-04-23

**Authors:** Martina Strittmatter, Laura J. Grenville‐Briggs, Lisa Breithut, Pieter Van West, Claire M. M. Gachon, Frithjof C. Küpper

**Affiliations:** ^1^ The Scottish Association for Marine ScienceScottish Marine InstituteOban, ArgyllScotlandPA37 1QAUK; ^2^ Aberdeen Oomycete LaboratoryInstitute of Medical SciencesUniversity of AberdeenForesterhillAberdeenScotlandAB25 2ZDUK; ^3^ Department of Plant Protection BiologySwedish University of Agricultural SciencesAlnarp230 53Sweden; ^4^ Fachbereich BiologieUniversität KonstanzKonstanzD‐78457Germany; ^5^ OceanlabUniversity of AberdeenMain StreetNewburghScotlandAB41 6AAUK

**Keywords:** model brown alga, reactive oxygen species, vanadium‐dependent bromoperoxidase

## Abstract

Pathogens are increasingly being recognized as key evolutionary and ecological drivers in marine ecosystems. Defence mechanisms of seaweeds, however, have mostly been investigated by mimicking infection using elicitors. We have established an experimental pathosystem between the genome brown model seaweed *E* *ctocarpus siliculosus* and the oomycete *E* *urychasma dicksonii* as a powerful new tool to investigate algal responses to infection. Using proteomics, we identified 21 algal proteins differentially accumulated in response to *E* *u. dicksonii* infection. These include classical algal stress response proteins such as a manganese superoxide dismutase, heat shock proteins 70 and a vanadium bromoperoxidase. Transcriptional profiling by qPCR confirmed the induction of the latter during infection. The accumulation of hydrogen peroxide was observed at different infection stages via histochemical staining. Inhibitor studies confirmed that the main source of hydrogen peroxide is superoxide converted by superoxide dismutase. Our data give an unprecedented global overview of brown algal responses to pathogen infection, and highlight the importance of oxidative stress and halogen metabolism in these interactions. This suggests overlapping defence pathways with herbivores and abiotic stresses. We also identify previously unreported actors, in particular a Rad23 and a plastid–lipid‐associated protein, providing novel insights into the infection and defence processes in brown algae.

## Abstract 2

Macroalgal response to pathogen infection has mostly been studied by mimicking infection using elicitors. We have established a pathosystem between the genome model seaweed *E* *ctocarpus siliculosus* and the oomycete *E* *urychasma dicksonii* as a powerful new tool to investigate algal responses to infection via a combined approach of proteomics, qPCR and histochemial staining. Our data give an unprecedented global overview of brown algal response to pathogen infection, and highlight the importance of oxidative stress and halogen metabolism in these interactions.

## Introduction

Macroalgae represent important components of cold and temperate rocky shore communities. Comparable with other organisms, they are subject to numerous biotic stresses (Potin *et al*. 2002; Gachon *et al*. 2010). Those stressors include viruses, bacteria, fungi, oomycetes, chytrids and algae in the form of endo‐ or epiphytes. The exploration of pathogens in Rhodophyceae, Chlorophyceae and Phaeophyceae macroalgae is steadily growing and numerous interactions between algae and stressors have been described over the last two decades. Algal endophytism is a prominent example, with the pathosystem between the red alga *Chondrus crispus* and the endophyte *Acrochaete operculata* being especially well described. Whereas the sporophytic phase of *Chondrus* is susceptible to infection by the endophyte, the gametophyte phase is resistant due to a different cell wall composition (Bouarab *et al*. 1999; Weinberger *et al*. 2005). With the recent completion of the *C. crispus* genome (Collén *et al*. 2013), new lines of research could be developed on this pathosystem.

The brown algal endophytes *Laminariocolax aecidiodes* and *Laminarionema elsbetiae* infect several kelp species via zoospores that germinate on the host surface and then penetrate its host (Heesch & Peters 1999). The oomycete *Olpidiopsis porphyrae* and the chytrid *Pythium porphyrae* challenge the economic important seaweed *Pyropia* (*Porphyra*) (Uppalapati & Fujita 2000; West *et al*. 2006). Recently, it was shown that recognition and attachment of the pathogenic zoospore to its host most likely involve lectin–carbohydrate binding (Klochkova *et al*. 2012). Despite the increasing knowledge on macroalgal host–pathogen interactions, algal response and defence reactions to such pathogens on a molecular level are little studied. Moreover, both in brown and red algae the current knowledge of algal response to pathogen attack in many instances derives from short‐term (1 h time scale) experiments on simplified models with elicitors (e.g. Weinberger & Friedlander 2000; Küpper *et al*. 2001). In particular, algal cell wall‐derived oligosaccharides, free fatty acids, methyl jasmonate and bacterial lipopolysaccharides were shown to induce an oxidative burst in the kelp *Laminaria digitata* (Hudson) J.V. Lamouroux (Küpper *et al*. 2001, 2006a, 2009). This accumulation of reactive oxygen species (ROS) is considered both a direct anti‐microbial defence reaction and an activator of downstream defence mechanisms such as oxylipin accumulation and the production of halogenated secondary metabolites (reviewed by Weinberger 2007). Since their original identification, elicitors have widely been used to mimic infection in *Laminaria digitata* to gain a deeper understanding of the molecular processes underlying defence responses (Tonon *et al*. 2008; Cosse *et al*. 2009; Goulitquer *et al*. 2009). These studies suggested a role for aldehyde accumulation in algal defences and identified defence‐related transcripts. Among the latter, vanadium‐dependent haloperoxidases were prominent and showed a tight regulation of expression. Despite the important defence and signalling role of ROS in *Laminaria digitata*, it appears that ROS‐independent signal transduction also occurs during the defence response (Cosse *et al*. 2009). The elicitor‐based defence response evokes a priming effect in *Laminaria digitata* (Thomas *et al*. 2011). Attacked individuals send out waterborne cues that condition neighbouring plants for a faster gene induction.

The capability to induce an oxidative burst by oligoguluronate elicitation varies between different brown algal species and notably is restricted to the sporophytic life stages of the Laminariales (Küpper *et al*. 2002). Whether similar signal transduction pathways occur in brown algal species where no oxidative burst has been observed is currently unknown and it appears plausible that those species might have different defence pathways (Zambounis *et al*. 2013).

One of those species is the filamentous seaweed *Ec. siliculosus* (Dillwyn) which, over the last few years, has emerged as brown algal genome model, culminating in the recently completed genome project (Cock *et al*. 2010, 2012). The development of tools such as microarrays and proteomics enables a comprehensive analysis of changes at the transcriptional and protein level. Importantly, this alga is host to the obligate‐biotrophic oomycete pathogen *Eurychasma dicksonii* (E.P. Wright) Magnus.

**Figure 1:**
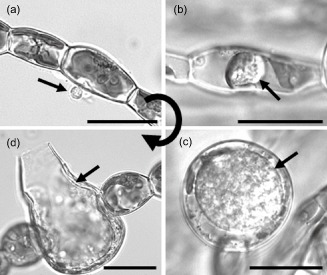
Life cycle of the intracellular oomycetes pathogen *E* *urychasma dicksonii* in its brown algal host *E* *ctocarpus siliculosus*. A spore (arrow) attaches to the algal surface and injects its content into the host (a). Within the algal cytoplasm, the *E* *u. dicksonii* thallus (arrow) develops which at the early stage of infection is unwalled (b). At a later stage, the pathogen thallus (arrow) has a cell wall and causes hypertrophic expansion of the algal host cell (c). At the final stage, the complete thallus differentiates into a sporangium from which motile zoospores (arrow) are produced completing the life cycle of the pathogen (d). Scale bars equal to 25 *μ*m.

In coastal ecosystems, *Eu. dicksonii* has been recorded in epidemic outbreaks throughout the world, and an exceptionally broad host spectrum of over 40 algal species is documented (Küpper & Müller 1999; Gachon *et al*. 2009; Strittmatter *et al*. 2009, 2013). It is an intracellular pathogen for most of its life cycle, and in contrast to eucarpic counterparts, infects single algal cells (Fig. 1a–d). It represents one of the very few eukaryotic algal pathogens that can be cultivated under laboratory conditions. Therefore, the *Eu. dicksonii–Ec. siliculosus* pathosystem is among the very few marine algal models amenable to molecular investigation (Gachon *et al*. 2009; Grenville‐Briggs *et al*. 2011). *Eu. dicksonii* is particularly interesting due to its basal phylogenetic position in the oomycete tree (Küpper *et al*. 2006b; Sekimoto *et al*. 2008), many of which are devastating pathogens in aquaculture (e.g. *Saprolegnia*, *Aphanomyces*) and agriculture (e.g. *Phytophthora*, *Plasmopara*, *Pythium*).

Yet, so far, the brown algal response to pathogen infection has never been investigated on a real pathosystem using a comprehensive, global ‘omics’ approach. In this context, we chose to investigate the interaction between *Ec. siliculosus* and *Eu. dicksonii* via 2‐DE‐based comparative proteomics and histochemical studies with the aim of identifying the cellular processes involved in the disease, and the algal responses to biotic stress. Proteomic tools have already been used in a number of studies investigating host–pathogen interactions in various systems like angiosperms and vertebrates including humans (e.g. Kav *et al*. 2007; Bhadauria *et al*. 2010; Roy *et al*. 2010; Schmidt & Völker 2011). In contrast to transcriptomic analysis, proteomics reflect more closely the abundance or activity of effector molecules responsible for a given phenotype (Tan *et al*. 2009).

In the present study, we present novel insights derived from a real host–pathogen interaction and show the involvement of ROS and putative halogen metabolism as response of *Ec. siliculosus* to pathogen infection.

## Materials and Methods

### Biological material and inoculation

Monoeukaryotic, axenic *Ec. siliculosus* CCAP 1310/4 (clonal male parthenosporophyte), *Macrocystis pyrifera* CCAP 1323/1 (clonal female gametophyte) and *Eu. dicksonii* CCAP 4018/1 were maintained as previously described in 650 mL filter cap suspension flasks (Greiner Cellstar; Müller *et al*. 2008). They were transferred into fresh medium every 3 weeks and monitored on a weekly basis by microscopy.

**Figure 2:**
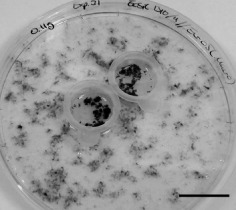
Experimental set‐up for the mass inoculation for the proteomic investigation of *E* *urychasma dicksonii*‐infected *E* *ctocarpus siliculosus.* *M* *acrocystis pyrifera* was used as alternate host of *E* *u. dicksonii* in cell strainers which allowed the free passage of pathogen spores into the surrounding medium containing the target alga *Ec* *. siliculosus.* Scale bar 30 mm.

For the proteomic investigation 50 mg fresh weight (FW) of *Ec. siliculosus* was distributed into 140 mm diameter Petri dishes containing 110 mL of one‐half strength modified Provasoli‐enriched natural seawater (PES; West & McBride 1999). The *M. pyrifera* female gametophyte was chosen as a convenient alternate host for the mass production of *Eu. dicksonii* inoculum because it is highly susceptible to the oomycete pathogen and does not produce any motile zoospores. Infected *M. pyrifera* was placed in two 70 *μ*m pore‐sized cell strainers (Falcon), a set‐up that avoids contamination of the target inoculated alga (here *Ec. siliculosus*) with infected inoculum (Fig. 2). The progress of infection was regularly monitored using an inverted bright field microscope. Controls were performed to check that the cell strainers allowed the free passage of *Eu. dicksonii* spores into the surrounding medium containing *Ec. siliculosus* (Gachon *et al*. 2009). Mock‐inoculated controls (uninfected *Ec. siliculosus*) were prepared in parallel by co‐incubating uninfected *M. pyrifera* with 50 mg of *Ec. siliculosus*. Throughout the incubation time 80% of the medium was replaced on a 3 week basis in order to avoid nutrient depletion.

The algal material was harvested when the highest possible density of symptoms was observed (approximately 1 algal cell in 10 infected, typically after 6–8 weeks). *Ec. siliculosus* was soaked dry on filter paper, weighed, frozen in liquid nitrogen and stored at −80 °C. Four Petri dishes of either *Eu. dicksonii*‐ or mock‐inoculated algal material were pooled yielding 180–200 mg (FW) for each of the mock and infected samples. In total three independent experiments were performed.

### Protein extraction, quantification and clean‐up for two‐dimensional electrophoresis

The protein extraction protocol developed by Contreras *et al*. (2008) was optimized to increase protein yield, as detailed in Supporting Information Table S1. Proteins were quantified using the 2‐D Quant™ kit (GE Healthcare, UK) according to the instruction manual (standard procedure). The protocol is based on the precipitation of polypeptides prior to quantification in order to remove interfering substances. Quantification is achieved by colorimetric detection of unbound cupric ions. The overall accuracy of the quantification was improved by including an additional precipitation step before adding the copper solution to the pellet.

The quality of the protein extracts was further checked by loading equal amounts (20 *μ*g) on a precast 4–20% polyacrylamide gel (Pierce, Rockford, IL, USA; Thermo Scientific) and subsequent staining with GelCode Blue stain (Pierce, Thermo Scientific). Prior to isoelectric focussing (IEF), 150 *μ*g of total protein extracts was cleaned by precipitation to reduce interfering substances such as polysaccharides and salts using the 2‐D clean‐up kit (GE Healthcare) according to the instruction manual (procedure for 1–100 *μ*L of sample volume). Finally, the protein pellet was resuspended in 25 *μ*L resuspension buffer [7 m urea, 2 m thiourea, 4% (w/v) CHAPS, 0.065 m DTT, 0.5% (v/v) Triton X‐100, 1% (w/v) carrier ampholytes; 100× Bio Lyte 3–10 buffers; Bio‐Rad, Hercules, CA, USA] and incubated for 30 min at room temperature.

### Two‐dimensional electrophoresis

A total of 125 *μ*L of rehydration buffer [7 m urea, 2 m thiourea, 2% (w/v) CHAPS, 0.018 m DTT, 0.5% (w/v) carrier ampholytes (100× Bio‐Lyte™ 3–10 buffer, Bio‐Rad), 0.002% (v/v) bromophenol blue solution] was added to the protein solution, briefly mixed and centrifuged at 16 000× *g* for 5 min in order to pellet any indissoluble matter. A 7 cm immobilized pH gradient (IPG) strip pH 4–7 (ReadyStrip, Bio‐Rad) was incubated with the supernatant in a rehydration tray for 1 h at room temperature and then overlaid with 1 mL of mineral oil. IPG strips were then passively rehydrated at RT for a total of 15 h. Afterwards the strips were placed on electrode wicks (humidified with 10 *μ*L MQ‐H_2_O Bio‐Rad) in a focusing tray and overlaid with 1 mL mineral oil. Proteins were focused on a Protean 3 IEF cell (Bio‐Rad) using the following successive protocol (rapid voltage ramp method; max. 50 *μ*A strip^−1^; 20 °C): step 1: 0–250 V for 125 Vhr; step 2: 250–4000 V for 4000 Vhr; step 3: 4000 V for 120 000 Vhr.

Three technical replicates were run per protein extract. Prior to running the second dimension (SDS‐PAGE), the IPG strips were incubated in equilibration buffer [0.05 m Tris/HCl pH 8.8, 6 m urea, 30% (v/v) glycerol, 2% (w/v) SDS, 0.002% (v/v) bromophenol blue solution] containing 10 mg mL^−1^ DTT for 25 min and subsequently in equilibration buffer containing 25 mg mL^−1^ iodoacetamide for 15 min. The equilibrated strips were then placed on a single well 12% Bis‐Tris Mini gel (NuPAGE Novex, Invitrogen, Carlsbad, CA, USA) and fixed with 0.35% (w/v) molten agarose NA (GE Healthcare, UK). Electrophoresis was performed in MOPS‐SDS running buffer (NuPAGE, Invitrogen) with a two‐step protocol (70 V for 30 min; 200 V for 55 min). Next, gels were fixed in 50% (v/v) MeOH, 7% (v/v) acetic acid for 10 min under shaking, followed by two washing steps in MQ‐H_2_O and were then incubated in GelCode Blue stain (Pierce, Thermo Scientific, USA) overnight. Gels were washed in MQ‐H_2_O to remove unbound stain and were scanned on an ImageScanner III (GE Healthcare, UK) using a resolution of 600 dots per inch (transparent mode, bits depth: 16 bits per channel, green filter). Gels were preserved by incubation in 0.5% (v/v) glycerol for 1 h with subsequent drying in cellophane until spot cutting.

### Image analysis

Image alignment, background subtraction and normalization were automatically performed using the Progenesis SameSpot™ software v3.0 (Nonlinear Dynamics, UK). Spot alignment and detection were manually improved as necessary. Spot densities were expressed as mean normalized volumes and fold changes between infection and control material were calculated. Based on the program's statistical analysis (one‐way analysis of variance), spots with a *P*‐value <0.05 and a fold change of ≥1.5 were selected for subsequent mass spectrometry.

In total, nine 2‐DE gels of control protein extracts and 2‐DE gels of infection protein extracts were analysed originating from three independent experiments.

### In‐gel digestion and liquid chromatography‐tandem mass spectrometry (LC‐MS/MS)

Spot cutting and the tryptic digestion of proteins and LC‐MS/MS analysis was performed as previously described (Grenville‐Briggs *et al*. 2005, 2008, 2010).

Peptide peaks were detected and deconvoluted automatically using the DataAnalysis™ software (Bruker, USA). Mass lists in the form of Mascot Generic Files were created automatically and used as the input for Mascot MS/MS Ions searches of the NCBI nr database and the *Ec. siliculosus* protein database (version ‘Ectsi_Prot_latest’ Cock *et al*. 2010) on the Matrix Science web server (www.matrixscience.com). The default search parameters used were as follows: enzyme = trypsin, max missed cleavages = 1; fixed modifications = carbamidomethyl (C); variable modifications = oxidation (M); peptide tolerance ± 1.5 Da; MS/MS tolerance ± 0.5 Da; peptide charge = 2 + and 3 + ; instrument = ESI‐TRAP. Probability‐based MOWSE scores above the threshold value (*P* < 0.05) were considered for protein identification. The mass spectrometry proteomics data and accompanying experimental conditions have been deposited to the ProteomeXchange Consortium (http://proteomecentral.proteomexchange.org) via the PRIDE partner repository (Vizcaíno *et al*. 2013) with the dataset identifier PXD001458.

### RNA extraction and transcriptional analysis via quantitative real‐time PCR

As for the proteomics analysis, *Eu. dicksonii*‐infected *Ec. siliculosus* tissue was harvested for subsequent RNA extraction once a dense infection in the late thallus stage was visible. Extraction of RNA, DNaseI treatment and cDNA synthesis was performed as previously described (Zambounis *et al*. 2013).

Oligonucleotides targeting selected genes were designed using the Beacon Designer (Premier Biosoft) for SYBR Green PCR assays with the following parameters T_annealing_ 60 ± 1 °C; length: 18–24 bp; amplicon length: 75–200 bp. A BlastN search of the primer sequences against the *Ec. siliculosus* nucleotide database (Cock *et al*. 2010) and a local *Eurychasma* EST database (Grenville‐Briggs *et al*. 2011) was performed to rule out possible cross‐reactions between genes. Their sequences are given in Supporting Information Table S2.

Real‐time PCR was run in three technical replicates (20 *μ*L each), with 1× Mesa Green Mastermix (Eurogentec, Seraing, Belgium), 300 nM of each primer and 2 ng of cDNA described in Zambounis *et al*. (2013). Relative expression ratios of tested genes were normalized against the constitutive reference gene EEF1*α*2 (Le Bail *et al*. 2008; Dittami *et al*. 2009). The relative expression ratios were calculated based on the PCR efficiencies and the crossing point [C(t) values] differences of each sample versus a mock‐treated (control) sample using the geometric mean of the technical replicates. The statistical significance of the results was tested with the pairwise fixed reallocation randomization test (2000 iterations) implemented in the software REST 2009 (Pfaffl *et al*. 2002). Whether a gene was significantly induced during any one treatment was judged by calculating the probability [P(H1)] that the observed difference between the sample and control groups was due only to chance [P(H1) < 0.05]. Five independent biological experiments were performed. Each encompassed two treatments (infected and mock‐inoculated control).

### Detection of ROS

In order to detect the accumulation of hydrogen peroxide in *Eurychasma*‐infected *Ectocarpus* tissue, the cultures were incubated in 3,3‐diaminobenzidine (DAB) dissolved in PES at a concentration of 1 mg mL^−1^ overnight at 15 °C in the dark. The following day, the samples were destained two times in 75% (v/v) ethanol and once in 100% (v/v) methanol for 5 min at 95 °C and observed by differential interference contrast (DIC) microscopy. Images were taken with an AxioCam™ HR3 (Zeiss, Germany). For the detection of superoxide radicals (O_2_^–^), DAB was replaced by nitrotetrazolium blue (NBT) at a final concentration of 1 mg mL^−1^. The superoxide dismutase inhibitor diethyldithiocarbamate (DDC) was used as previously described (Küpper *et al*. 2001). Briefly, uninfected and *Eu. dicksonii*‐infected algal tissue was incubated in 1 mm DDC (from a 100 mm stock solution dissolved in ethanol) for 3 h at 15 °C in the dark before addition of NBT or DAB as described earlier. As positive controls, uninfected *Ec. siliculosus* was incubated under medium light (30 *μ*E m^−2^ s^−1^). Experiments were performed at least three times independently.

## Results

### Improved protein extraction protocol and protein map of uninfected *Ec* *. siliculosus*

Brown seaweeds are a challenging biological material for protein extraction, typically leading to rather poor yields. Due to the intracellular nature of the pathogen, the amount of infected tissue was limiting to perform the proteomics analysis. Therefore, a previously established protocol (Contreras *et al*. 2008) was modified (detailed in Supporting Information Table S1), leading to increased protein yields of 2–10 *μ*g mg^−1^ FW compared with 0.5–1 *μ*g mg^−1^ FW with the initial protocol. Hence, a total amount of 200 mg algal material was sufficient to run at least three 7 cm 2‐DE gels (150 *μ*g total protein per gel).

Approximately 600 *Ec. siliculosus* proteins were resolved by 2‐DE. In order to obtain an overview of the proteins expressed in healthy *Ec. siliculosus* tissue, 26 protein spots from control gels were selected across acidic, basic, high and low molecular weight ranges and were subjected to LC‐MS/MS analysis (Supporting Information Fig. S1a). All proteins were matched to *Ec. siliculosus* sequences (Supporting Information Table S3). They represent a broad spectrum of housekeeping proteins expected to be expressed in healthy, uninfected *Ectocarpus* cells, such as photosynthesis‐related proteins (e.g. light‐harvesting complex protein, cytochrome b_6_f complex and ferredoxin‐NADP oxidoreductase). Furthermore, several enzymes involved in primary metabolism were identified, for example, pyruvate carboxylase (regeneration of citric acid cycle metabolites), glyceraldehyde‐3‐phosphate dehydrogenase (Calvin cycle), transketolase (Calvin cycle and oxidative pentose pathway) and triosephosphate isomerase/glyceraldehyde‐3‐phosphate dehydrogenase (glycolysis). Note that several of these proteins are integral to the membrane (e.g. light‐harvesting complex protein, cytochrome b_6_f complex) indicative of efficient protein recovery and good overall representation.

### Ectocarpus proteome profiling in response to *E* *urychasma* infection

The densely infected *Ec. siliculosus* biomass contained mostly intracellular pathogenic thalli and a few mature dehiscent *Eu. dicksonii* sporangia (Fig. 1c,d). During this stage, the host nucleus and microtubules are still intact (Tsirigoti *et al*. 2013, 2014), and a therefore an active host response can be monitored. The comparison of protein profiles revealed 21 spots whose relative abundance varied significantly between healthy and infected *Ec. siliculosus* (Supporting Information Figs S1b & S2). Among these, 18 protein spots showed a significantly higher expression compared with the control and three protein spots a lower expression (Table 1). All spots were attributed to *Ec. siliculosus* proteins, in agreement with the observation that no newly expressed protein spots were detected from the gels of infected algal material.

**Table 1:** Differentially expressed protein spots of *E* *ctocarpus siliculosus* CCAP 1310/4 during infection with *E* *urychasma dicksonii* CCAP 4018/1

Protein spot ID^a^	*Ectocarpus siliculosus* accession code^b^	Protein identification^c^	Fold change (*P*‐value^d^)	Number of peptides matching	Sequence coverage (%)	Theoretical MW (Da)	Observed/theoretical pI	Mascot score^e^
Energy/metabolism								
67^f^	Esi0199_0054	Light harvesting complex protein	**↑** 1.6 (7.8e^−05^)	1	3	28 905	4.52/4.66	38
74	Esi0128_0015	Endo‐1,3‐*β*‐glucanase, family GH17	**↓** 1.6 (7.4e^−06^)	3	15	36 052	6.24/5.77	63
112^f^	Esi0000_0413	Dihydrolipoamide dehydrogenase	**↓** 1.5 (3.2e^−06^)	2	4	53 496	6.13/6.16	50
Defence and cell rescue								
23^f^	Esi0091_0024	Manganese superoxide dismutase	**↑** 1.9 (1.5e^−05^)	4	12	32 055	5.61/6.34	79
24^f^	Esi0009_0080	Vanadium‐dependent bromoperoxidase	**↑** 1.9 (2.8e^−07^)	36	39	70 583	6.3/5.914	750
46^f^	Esi0116_0066	Plastid lipid‐associated protein	**↑** 1.7 (6.15e^−08^)	17	33	47 238	4.72/4.95	611
48^f^	Esi0162_0003	Aspartyl protease	**↑** 1.7 (1.5e^−06^)	15	48	44 354	4.6/4.84	459
53^f^	Esi0116_0066	Plastid lipid‐associated protein	**↑** 1.7 (5.6e^−06^)	8	21	47 238	4.6/4.95	269
Protein synthesis, folding and turnover								
11^f^	Esi0017_0100	Rad23‐like protein UV excision repair protein	**↑** 2.2 (2.9e^−11^)	7	15	47 631	4.76/4.57	243
31^f^	Esi0241_0010	Heat shock protein 70	**↑** 1.8 (1.4e^−07^)	4	5	59 614	5.76/5.63	86
33	Esi0002_0284	Heat shock protein 70	**↑** 1.8 (8.6e^−05^)	27	43	72 322	4.82/4.72	595
54^f^	Esi0073_0091	Elongation factor 1Bу	**↓** 1.7 (2.8e^−06^)	9	26	47 728	6.4/6.2	108
66	Esi0164_0066	Protein disulfide isomerase	**↑** 1.6 (2.5e^−04^)	28	45	53 685	5.03/4.95	686
120	Esi0164_0064	Chaperonin	**↑** 1.5 (1.1e^−05^)	23	45	66 144	4.95/4.96	602
Unknown/hypothetical proteins								
16^f^	Esi0209_0047	Endonuclease/exonuclease/phosphatase domain	**↑** 2 (5.4e^−05^)	1	4	23 411	6.2/5.29	46
36^f^	Esi0125_0013	Chloroplast protein EST support	**↑** 1.7 (2.2e^−07^)	6	26	41 340	4.72/4.91	139
44	Esi0196_0051	Anion‐transporting ATPase domain	**↑** 1.7 (5.4e^−06^)	1	4	25 392	4.54/5.49	33
61^f^	Esi0077_0002	PAP fibrillin domain	**↑** 1.6 (4.2e^−06^)	10	30	23 348	4.54/4.72	180
80^f^	Esi0002_0309	NTF2‐like domain	**↑** 1.6 (2.2e^−06)^	7	31	27 988	4.85/5.27	185
88^f^	Esi0282_0008	Nascent polypeptide‐associated complex (*α* subunit) domain	**↑** 1.5 (3.7e^−07^)	8	55	20 662	4.6/4.48	331
130	Esi0125_0013	Chloroplast protein EST support	**↑** 1.4 (1.8e^−05^)	8	32	41 340	4.76/4.91	309

a Rank Progenesis SameSpots (representative 2‐DE gel in Supporting Information Figs S1b and S2).

b *Ectocarpus siliculosus* genome.

c Based on the most significant match (Mascot).

d One‐way analysis of variance (Progenesis SameSpots).

e Individual ions scores >33 indicate identity or extensive homology (*P* < 0.05).

f Differential expression in three independent experiments.

↑, increased abundance as compared with the control; ↓, decreased abundance as compared with the control; MW, molecular weight; PAP, plastid–lipid‐associated protein; pI, isoelectric point.

The spot with the highest differential expression (spot ID 11; fold change 2.2) had highest matches to a Rad23‐like protein/UV excision repair protein. Two protein spots (spot ID 23 and 24) with a 1.9‐fold induction in infected material were attributed to a manganese superoxide dismutase (MnSOD) and a vanadium‐dependent bromoperoxidase (vBPO), respectively. Other noticeable induced proteins were members of the heat shock protein 70 (HSP70) family [spot ID 31 and spot ID 33 (HSP70_1)], a disulfide isomerase (spot ID 66), a chaperonin (spot ID 120) and an aspartyl protease (spot ID 48). Interestingly, several plastidial proteins were induced: a plastid–lipid‐associated protein (PAP) matched two spots (spot IDs 46 and 53) with slightly different isoelectric points, which suggests different posttranslational modifications (PTM) as well as another PAP/fibrillin domain‐containing protein (PAPfib, spot ID 61). Different PTMs might also be attributed to spot IDs 36 and 130, which match to a chloroplast‐localized protein of *Ectocarpus* that is highly supported by EST data (Cock *et al*. 2010) but does not have any conserved domains. Spot ID 88 showed a positive fold change of 1.5; the conserved domain within this protein was assigned to the *α*‐subunit of a nascent polypeptide‐associated complex.

The three protein spots identified as repressed during infection matched a component of the eukaryotic elongation factor 1B (EEF 1B) complex (spot ID 54), a dihydrolipoamide dehydrogenase (spot ID 112) and a GH17 endo‐1, 3‐*β*‐glucanase (spot ID 74).

### Transcript profiling of selected stress marker genes by qPCR

**Figure 3:**
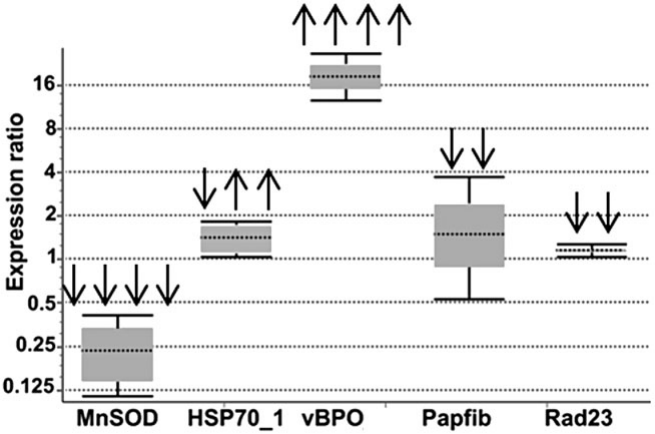
Transcription profiling of five *E* *ctocarpu*s genes: manganese superoxide dismutase (MnSOD), heat shock protein family 70 (HSP70_1), vanadium‐dependent bromoperoxidase (vBPO), plastid lipid‐associated/fibrillin (Pabfib) and Rad 23‐like (Rad23) encoding for differentially accumulated proteins during pathogen infection. The relative expression ratios are shown as whisker box plots showing the median and extreme expression ratios of the respective genes. The diagram gives the result of one representative experiment with three technical replicates. Arrows above the box plots indicate the number of independent experiments (out of five) in which the gene showed significant up‐regulation (↑) or significant down‐regulation (↓) at *P* < 0.05.

Five genes encoding proteins differentially expressed during infection were selected for transcriptional profiling: vBPO, MnSOD, HSP70_1, Papfib and Rad23 (Fig. 3).

The vBPO gene was significantly up‐regulated (*P* < 0.05) in 4 out of 5 independent experiments and showed a fold change from 7.5 to 35. HSP70_1 was induced in two independent experiments (fold change from 1.4 to 16) but repressed in one experiment (fold change 0.5). In contrast to our proteomics data, transcript levels of MnSOD were significantly reduced in four experiments (expression ratio between 0.2 and 1.4). The remaining two genes Rad23 and Pabfib were down‐regulated in two independent samples with a fold change between 0.07 and 0.9 for Rad23 and 0.18 and 0.27 for Pabfib, whereas in the remaining three independent experiments those two genes were not differentially expressed.

### Infected *Ec* *. siliculosus* and *E* *u. dicksonii* produce ROS

**Figure 4:**
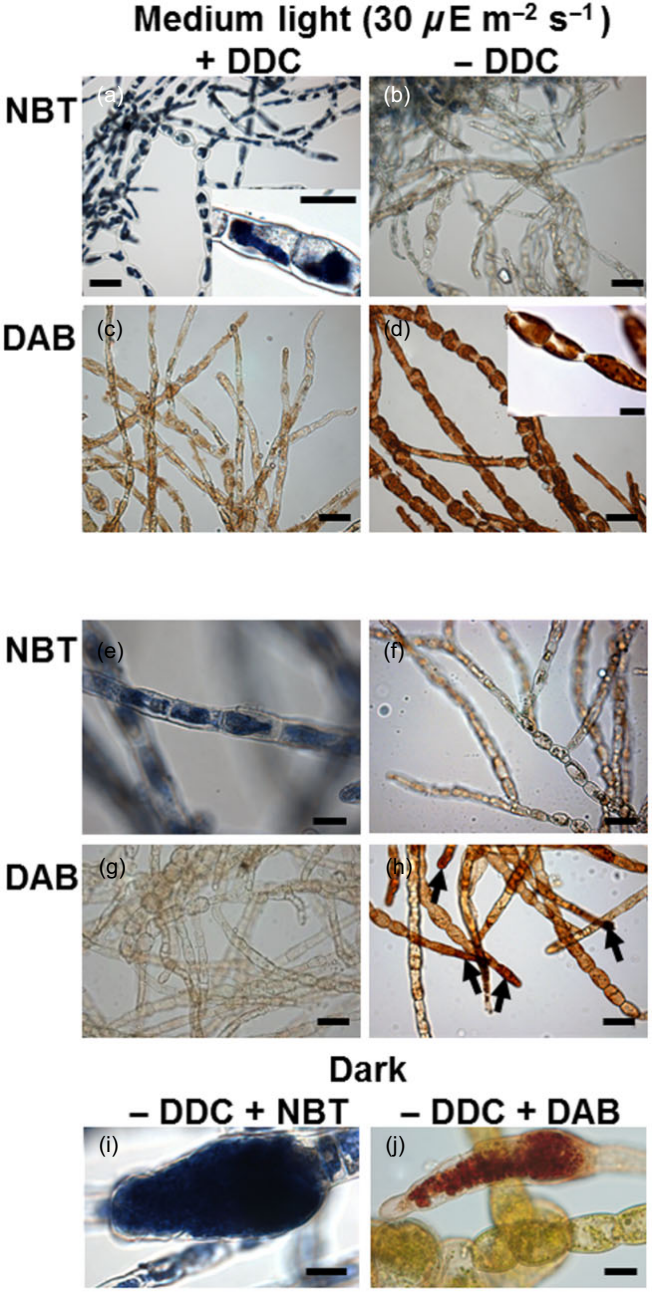
Histochemical detection of H_2_ O_2_ and O_2_^–^● in uninfected *E* *ctocarpus siliculosus* controls incubated in medium light (30 *μ*E m^−2^ s^−1^; a–d) or in the dark (e–j). Scale bars equal to 20 *μ*m in all pictures.

Because the proteomic analysis revealed two proteins, MnSOD and vBPO, potentially involved in the detoxification of ROS, we further investigated the presence of ROS in infected tissue via DAB and NBT histochemical staining. The former compound is oxidized in the presence of hydrogen peroxide (H_2_O_2_) by peroxidases, forming a brown precipitate. The latter is reduced in the presence of superoxide radicals (O_2_^–^●), forming a blue precipitate. DDC is an inhibitor of SOD that catalyses the conversion of O_2_^–^● into H_2_O_2_. As a positive control, we checked that inhibition of SOD with DDC leads to a build‐up of O_2_^–^● in the chloroplasts of uninfected *Ec. siliculosus* cells during exposure to moderate light (30 *μ*E m^−2^ s^1^, Fig. 4a), whereas only a weak accumulation of O_2_^–^● is detected in the absence of DDC (Fig. 4b). Conversely, a systemic plastidial generation of hydrogen peroxide is detected with DAB in those samples (Fig. 4d), and lost in DDC‐treated samples (Fig. 4c).

A weaker, yet still detectable build‐up of O_2_^–^● is likewise observed in dark‐incubated, DDC‐treated, uninfected algal tissue (Fig. 4e). In the absence of DDC, the observed superoxide build‐up is even less, in agreement with the reported short life span of this free radical (Fig. 4f). In contrast, the build‐up of H_2_O_2_ in dark‐incubated, DDC‐untreated and uninfected *Ec. siliculosus* was predominantly restricted to apical cells (Fig. 4h). In the corresponding DDC‐treated controls, H_2_O_2_ could not be detected at all (Fig. 4g). *Ec. siliculosus* mitospores within plurilocular sporangia were also a light‐independent source of ROS (Fig. 4i,j).

**Figure 5:**
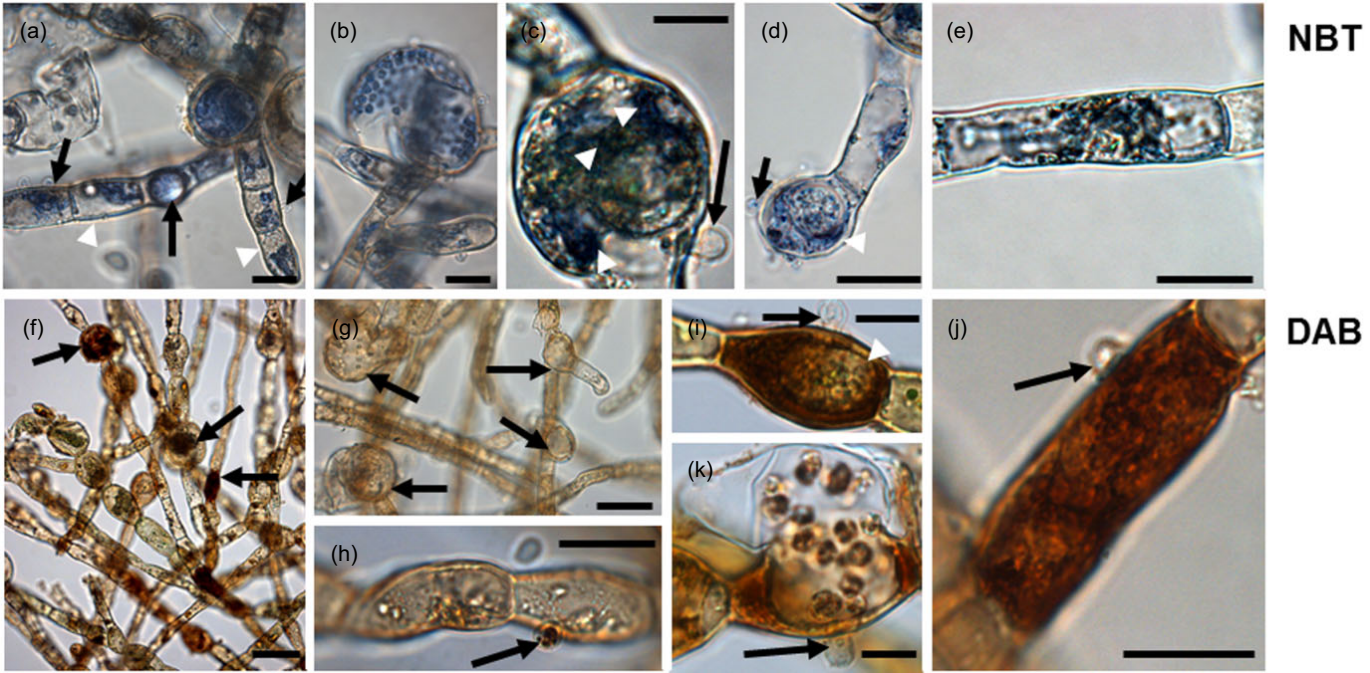
Detection of the reactive oxygen species superoxide by the histochemical stain nitrotetrazolium (a–e) and 3,3‐diaminobenzidine (f–k) in *E* *urychasma dicksonii*‐infected *E* *ctocarpus siliculosus*. (a) Constitutive, global superoxide generation in *Ec* *. siliculosus*. Cells that have been challenged with the pathogen *E* *u. dicksonii* are marked with black arrows and uninfected cells with white arrowheads. (b) Superoxide generation in secondary zoospores within a sporangium of the pathogen *E* *u. dicksonii*. (c, d) Blue labelling is visible around the pathogen thallus in the algal cytoplasm (white arrowheads). Arrows indicate the *E* *u. dicksonii* spore at the host surface. The pathogen thallus itself produces superoxide in some instances (d), blue labelling within the pathogen thallus structure). (e) Cytoplasmic labelling of an unchallenged host cell within an infected filament. (f) The production of H_2_ O_2_ is limited to infected and apical algal cells (arrows) in SOD‐uninhibited samples. (g) In diethyldithiocarbamate‐treated, *E* *u. dicksonii*‐infected *Ectocarpus* tissue 3,3‐diaminobenzidine does not precipitate in challenged cells (arrows). (h) Initial infection stage. The pathogen spore is attached to the algal surface (arrow) but the pathogenic cytoplasm has not been injected into the host cytoplasm. (i, j) Thallus phase of *E* *u. dicksonii*. At this stage, the pathogen spores at the host surface (arrows) are empty. White arrowheads denote the pathogen thallus. (k) Late sporangium stage. *E* *urychasma* secondary zoospores within the sporangium are also stained. Scale bars equal to 20 *μ*m in a, b, d, e, h and j; to 10 *μ*m in c; and to 50 *μ*m in f and g.

Infection triggered a mild increase in the light‐independent superoxide accumulation on top of the constitutive production described earlier (Fig. 5a). O_2_^–^● was generally produced in *Eu. dicksonii* secondary zoospores within the pathogen sporangium (Fig. 5b). During earlier, intracellular infection stages, NBT staining of infected algal cells was more heterogeneous. Algal‐derived blue labelling was sometimes observed around young walled *Eu. dicksonii* thalli (Fig. 5b–d), but also within the thalli (Fig. 5d), indicating that during this infection stage the alga as well as the pathogen may generate superoxide. In some instances, increased cytoplasmic superoxide production was also seen in unchallenged *Ec. siliculosus* cells (Fig. 5e).

*Eu. dicksonii*‐infected host cells were DAB positive (Fig. 5f), and the staining was totally abolished in the presence of DDC, indicating that superoxide dismutation is the source of H_2_O_2_ generation in response to infection (Fig. 5g). Despite some variability, typical patterns of H_2_O_2_ were apparent: before penetration, encysted *Eu. dicksonii* spores were labelled (Fig. 5h). Thereafter, the complete host cell content around the intracellular pathogen structures was most often stained (Fig. 5i,j). Overall, during the late thallus phase, H_2_O_2_ was highly present. Even when the developing pathogen sporangium had already filled the host cell, H_2_O_2_ was still produced by *Ec. siliculosus* (Fig. 5k). Consistent with NBT labelling, *Eu. dicksonii* secondary spores accumulate H_2_O_2_ (Fig. 5k).

## Discussion

**Figure 6:**
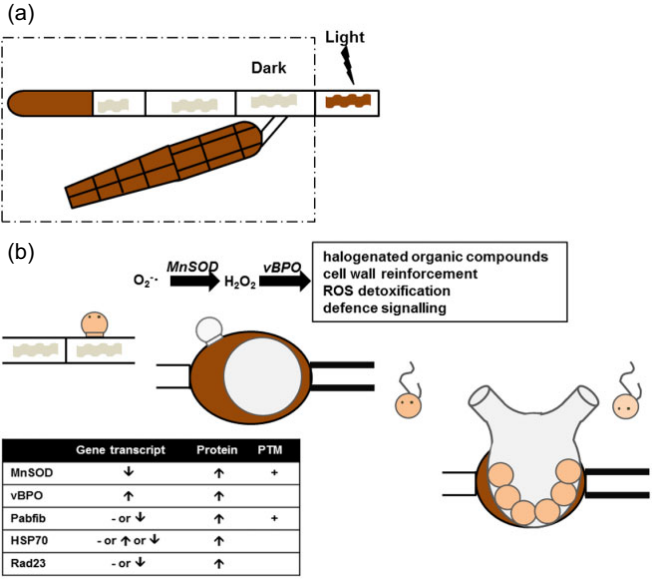
Working model of the defence reactions of *E* *ctocarpus siliculosus* during infection by *E* *u. dicksonii*. *Ec* *. siliculosus* and *E* *u. dicksonii* structures are outlined with black and grey lines, respectively. H_2_ O_2_ production is highlighted in brown; chloroplasts are shown as ribbon structures. (a) Constitutive, light‐independent H_2_ O_2_ generation in *Ec* *. siliculosus* tips and plurilocular sporangia, originating from the dismutation of O_2_^–^● radicals. Light‐dependent plastidial O_2_ production also leads to H_2_ O_2_ accumulation (dark‐brown undulated structures). (b) Cytoplasmic, light‐independent H_2_ O_2_ generation in the infected algal cell coincides with the accumulation of the MnSOD protein, and the strong transcriptional induction of vBPO, likely leading to reactive oxygen species detoxification, production of antimicrobial halogenated organic compounds and/or cell wall reinforcement. During zoosporogenesis, punctate cytosolic sources of O_2_^–^● and H_2_ O_2_ (probably mitochondria) are also observed in *E* *u. dicksonii.* ↑, significantly (*P* < 0.05) increased gene transcript or protein abundance; ↓, significantly (*P* < 0.05) decreased gene transcript or protein abundance; –, no change in gene expression; PST, posttranslational modification.

This study represents the first proteomic investigation of the biotic stress response in macroalgae. In contrast to previous investigations, this study is based on an actual host–pathogen interaction rather than mimicking infection via elicitor treatment. It reveals a complex, integrated picture of the response to infection that combines classical stress markers such as oxidative stress and halogen metabolism, with novel protagonists such as plastidial proteins (Fig. 6).

The maximum protein induction (2.2 fold change) detected between control and infected *Ec. siliculosus* was rather low compared with similar proteome studies on plant–pathogen interactions (e.g. Zhou *et al*. 2006). This is likely caused by a dilution effect in our protein samples as *Eu. dicksonii* infection is restricted to individual host cells and is asynchronous. Therefore, our proteomic results encompass a more localized and probably systemic response which recently has also been described in *Laminaria digitata* (Thomas *et al*. 2014). The highest fold change is close to the 1.5 cut‐off that we applied to detect significant changes in protein abundance, suggesting that our study underestimates the cohort of truly differentially accumulated proteins. Therefore, the application of mass spectrometry imaging methods (Matros & Mock 2013) is a tantalizing avenue to further the study of systemic response and localization of specific proteins during the course of infection.

As a result of this dilution, it is unsurprising that no oomycete proteins were detected by comparative 2‐DE.

### The *E* *ctocarpus* response to *E* *urychasma* infection

Several proteins identified in the course of this study have previously been reported as induced or differentially expressed by a range of abiotic stressors in macroalgae including *Ec. siliculosus* (Ritter *et al*. 2010), the kelp species *Laminaria digitata* (Cosse *et al*. 2009), *M. pyrifera* (Konotchick *et al*. 2013) and *Fucus* sp. (Pearson *et al*. 2010).

These proteins include HSP70s, which prevent aggregation and are major components of the protein folding process in a cell and are typical stress markers across phyla (Mayer & Bukau 2005). MnSOD is a classical ROS‐scavenging enzyme, which converts highly reactive superoxide into hydrogen peroxide. Among all proteins identified, vanadium‐dependent haloperoxidases are prominent brown algal stress markers in *Laminaria* and *Ectocarpus* (Crépineau *et al*. 2000; Roeder *et al*. 2005; Cosse *et al*. 2009). Significantly, our study constitutes the first direct evidence of a role for a vanadium haloperoxidase during pathogen infection. Based on the detection of the ROS H_2_O_2_ (a substrate for bromoperoxidase) in *Eurychasma*‐infected *Ectocarpus*, it is tempting to speculate on a role of the bromoperoxidase in scavenging of ROS and producing antimicrobial halogenated compounds. Our hypothesis is supported by the fact that expression of the bromoperoxidase also increases in *Ectocarpus* upon copper stress and elicitation with H_2_O_2_ and linolenic acid (Mithöfer *et al*. 2004; Ritter *et al*. 2010; Zambounis *et al*. 2013). A role of iodide and, to some extent, of bromide as antioxidants similar to *Laminaria* (Küpper *et al*. 2008, 2013) can be hypothesized, given the speciation of bromine and concentrations of both halogens in *Ectocarpus* established recently (Küpper *et al*. 2014). Furthermore, halogenated organic compounds are produced upon both oxidative and simulated biotic stress in *Laminaria* (Palmer *et al*. 2005; Goulitquer *et al*. 2009; Thomas *et al*. 2011), and have a demonstrated antimicrobial action in red and brown algae (Butler & Carter‐Franklin 2004; Paul *et al*. 2006; La Barre *et al*. 2010). VBPO, which has a predicted extracellular location (Cock *et al*. 2010), may also have a role in modifying the algal cell wall. It has recently been demonstrated that a bromoperoxidase is involved in the *in vitro* cross‐linking of phenolic substances and alginate in the presence of H_2_O_2_ (Salgado *et al*. 2009), and *Eu. dicksonii* infection triggers cell wall modifications (Tsirigoti *et al*. 2013, 2014). Even though the identification of a vBPO among the 21 differentially expressed proteins of *Ec. siliculosus* in response to infection with *Eu. dicksonii* suggests parallels to the elicitor‐based transcriptome studies in *Laminaria digitata* (Cosse *et al*. 2009), there are clear differences between the two brown algal species. In *Laminaria digitata* different members of the multigene haloperoxidase families (iodo‐ and bromoperoxidase) show differential expression under various stress conditions (Crépineau *et al*. 2000; Roeder *et al*. 2005; Cosse *et al*. 2009). Isoforms of these *Laminaria digitata* haloperoxidases show tightly coordinated expression in response to oligoguluronate elicitor treatment (Cosse *et al*. 2009), suggestive of functional specialization. In contrast to the kelp *Laminaria digitata*, the genome of *Ec. siliculosus* contains only one haloperoxidase gene encoding a vBPO (Cock *et al*. 2010), suggesting that halogen metabolism in *Ectocarpus* is less elaborate compared with kelp species. Ultimately, reverse genetic approaches, which are currently being developed in brown algae, will help to elucidate specific functions of this important protein family in *Laminaria* and *Ectocarpus* (Cock *et al*. 2012; Farnham *et al*. 2013).

Whether the presence of ROS produced by *Ectocarpus* in response to *Eurychasma* infection has a function in defence signal transduction and intracellular communication (Orozco‐Cárdenas *et al*. 2001) or in defence needs to be validated but data from *Laminaria* strongly suggest such a role (Cosse *et al*. 2009; Thomas *et al*. 2011). However, despite repeated attempts, we were unable to identify a trigger for *Eu. dicksonii* spore release, and to synchronize the infection of algal cells. Hence, we cannot investigate events that might occur within minutes after *Eu. dicksonii* adhesion or penetration in its host. Due to this limitation, we cannot rule out the possible occurrence of a transient H_2_O_2_ burst, fuelled by NADPH oxidase‐dependent superoxide production, as has been reported in *Laminaria digitata* (Küpper *et al*. 2002). Our data simply point to the existence of a sustained H_2_O_2_ source in infected algal cells throughout the development of the pathogen. We found a constitutive superoxide production in all *Ectocarpus* cells irrespective of the infection status, which only partially correlates with hydrogen peroxide accumulation in the dark. This indicates that whatever the light conditions, the algal cell type and the infection status, superoxide is by far the main source of H_2_O_2_ in *Ec. siliculosus*. However, in vegetative cells kept in the dark, there seem to be hydrogen peroxide detoxification routes (e.g. glutathione‐S‐transferase, glutaredoxin, catalase). Transcripts of classical ROS scavenger enzymes have been detected under abiotic stress conditions in *Ec. siliculosus* (de Franco *et al*. 2008; Dittami *et al*. 2009; Zambounis *et al*. 2013). It is also likely that the observed ROS generation upon high light stress and infection originates from different cellular compartments. Whereas during light stress the chloroplast is the primary source of ROS production (Asada 2006), the source during infection appears to be different. We could demonstrate that H_2_O_2_ accumulates preferentially in infected cells. This observation is in agreement with the increased protein abundance of MnSOD seen in our proteomics analysis. Additionally, the increased abundance of vBPO utilizing H_2_O_2_ during *Eu. dicksonii* infection would explain the increased ROS levels. As shown in *Laminaria digitata*, H_2_O_2_ might, however, not represent the only signal mediating defence responses (Cosse *et al*. 2009).

### Novel stress‐related proteins

In addition to the classic features of algal stress responses discussed earlier, our data also highlighted novel proteins that have not been described in the context of algal stress responses so far. A number of proteins (spot IDs: 53, 46, 36, 130 and 61) with increased abundance in *Ec. siliculosus* in response to infection have been predicted as chloroplast localized (Gschloessl *et al*. 2008). Among those, PAP/fibrillins appear as prominent stress proteins induced under various stress conditions such as high light, oxidative stress, drought and notably pathogen infection and wounding in higher plants (Langenkämper *et al*. 2001; Yang *et al*. 2006; Chen *et al*. 2007; Youssef *et al*. 2010). They constitute the main protein component of plastoglobules (lipoprotein structures) that are associated with the chloroplast thylakoid membrane via a half‐lipid bilayer in plants, and contain lipids and a variety of metabolic enzymes (Austin *et al*. 2006; Ytterberg *et al*. 2006; Bréhélin *et al*. 2007). In *Arabidopsis*, a knockdown of fibrillin expression leads to increased stress sensitivity including enhanced susceptibility to pathogens (Singh *et al*. 2010) and reduced accumulation of triacylglycerols and jasmonate (Youssef *et al*. 2010) but the exact functions are still unclear. Assuming that the increased expression of PAPs upon infection with *Eurychasma* signifies an accumulation of plastoglobules in *Ec. siliculosus*, the latter might either protect the chloroplast against increased levels of ROS (Vidi *et al*. 2006) or contribute to the elevated production of lipids and oxylipin precursors involved in defensive signalling (Santino *et al*. 2013). Further quantitative electron microscopy studies are warranted to investigate infection‐related plastoglobule accumulation. PAPs are often phosphorylated and differential phosphorylation of those proteins could explain the presence of two spots (ID 46 and ID 53) attributed to the same PAP protein in the present study. Differential phosphorylation may also explain the occurrence of two different spots (ID 36 and ID 130) as the same hypothetical, chloroplast‐located protein. Additionally, we found the MnSOD with slightly different isoelectric points. Whereas one spot (ID 724) showed unchanged abundance in response to biotic stress, a second protein spot (ID 23) assigned to the same protein had a lower isoelectric point of 5.61. The observation of potential posttranslational modification is indicative of regulation at the protein level rather than the gene expression level that is in agreement with our qPCR data on Papfib and MnSOD. Regulation at the posttranscriptional level allows a rapid response as it does not involve new protein biosynthesis and is commonly observed as response to pathogen attack in plants (Park *et al*. 2012).

The induction of a Rad23‐like protein in an infection‐related context was presented in two studies of the host response to infection with the protozoan pathogen *Toxoplasma gondii* in human cells (Nelson *et al*. 2008) and a plant pathogenic nematode *Meloidogyne* sp. (Fosu‐Nyarko *et al*. 2009). One important function of Rad23 proteins, demonstrated for example in yeast and *Arabidopsis*, is the association of polyubiquitinated proteins and the protein degradation machinery (26S proteasome) (Chen & Madura 2002; Dantuma *et al*. 2009; Farmer *et al*. 2010) via the two binding domains, ubiquitin‐associated and ubiquitin‐like of Rad23. In this context, the increased expression of a Rad23‐like protein in *Ec. siliculosus* in response to pathogen infection might signal increased protein degradation via the ubiquitin‐26S proteasome pathway that is in agreement with the increased abundance of folding proteins (e.g. HSP70, protein disulfide isomerase, nascent polypeptide‐associated complex protein, chaperonin) upon *Eu. dicksonii* infection.

In conclusion, the investigation of the *Ectocarpus*–*Eurychasma* host–pathogen interaction gives unprecedented insight into the algal host defence response, linking our results with previous studies on simulated biotic stress (Cosse *et al*. 2009; Zambounis *et al*. 2013). The final outcome of the interaction, however, is the successful invasion of the algal cell by the pathogen and therefore host defences, at least in the challenged cell, are ultimately overcome. Interestingly however, ROS are also detected during the late infection stages of *Eu. dicksonii* suggesting that the host response is still, at least partially, active. In this regard, the ongoing investigation of *Ectocarpus* strains resistant to *Eu. dicksonii* infection (Gachon *et al*. 2009) will shed more light on defence and immunity in macroalgae.

## Supporting information


**Figure S1.** Representative 2‐DE gels (pI 4–7) of uninfected *Ectocarpus siliculosus* (control, a) and *Eurychasma dicksonii*‐infected *Ectocarpus siliculosus* (infection, b). The indicated spot IDs match those of Table 1 (infection) and Supporting Information Table S3 (control).

**Figure S2.** Protein spots with significant (*P* < 0.05; one‐way anova) different abundance during *Eurychasma dicksonii* infection of the brown alga *Ectocarpus siliculosus*. The pictures show representative sections of control (C) and infection (I) of one 2‐DE gel.

**Table S1.** Modifications made to the original protocol (Contreras *et al*. 2008) for protein extraction of *Eurychasm*a *dicksonii*‐infected *Ectocarpus siliculosus*.

**Table S2.** Oligonucleotide sequence information of *Ectocarpus siliculosus* genes analysed by qPCR.

**Table S3.** Protein expression in uninfected *Ectocarpus siliculosus* CCAP 1310/4 (‘controls’).
