# Supplementary figures and images for: Infection of the brown alga E ctocarpus siliculosus by the oomycete E urychasma dicksonii induces oxidative stress and halogen metabolism
Source: Plant Cell Environ. 2015 Apr 23;39(2):259–71. doi: 10.1111/pce.12533 (PMC4949667; doi:10.1111/pce.12533)

Supplementary Figure 1

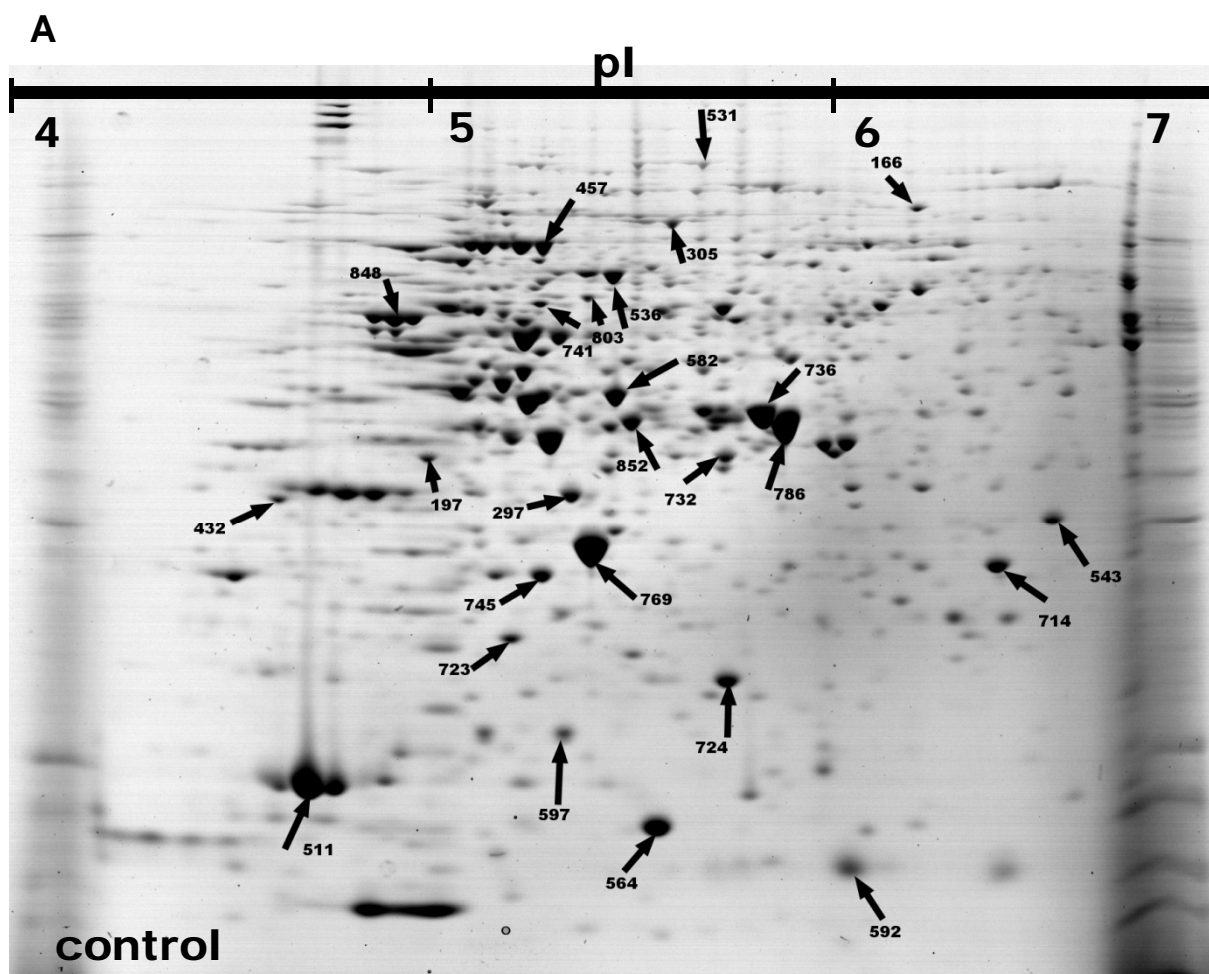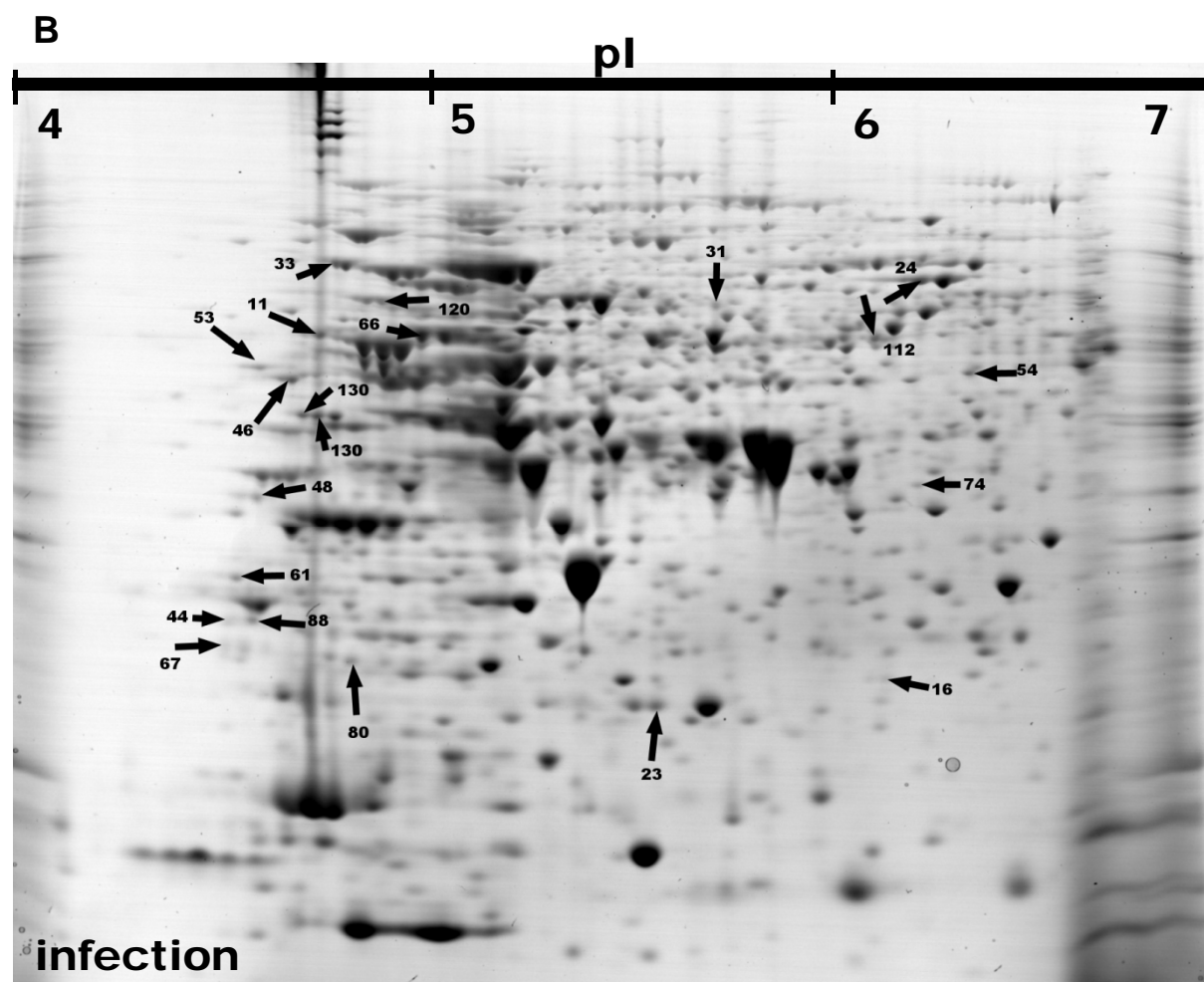

Supplement: Supplementary file 1 — Figure S1. Representative 2‐DE gels (pI 4–7) of uninfected Ectocarpus siliculosus (control, a) and Eurychasma dicksonii‐infected Ectocarpus siliculosus (infection, b). The indicated spot IDs match those of Table 1 (infection) and Supporting Information Table S3 (control). [file PCE-39-259-s001.pdf]

**Supplementary Figure 2**

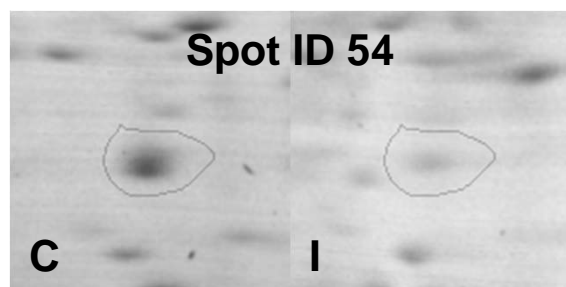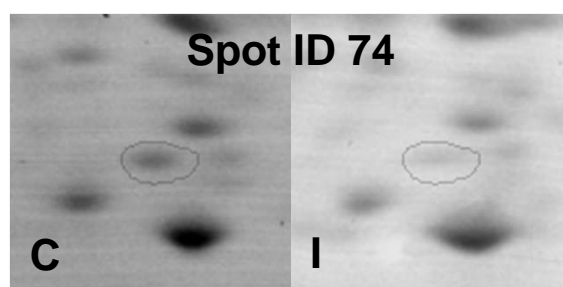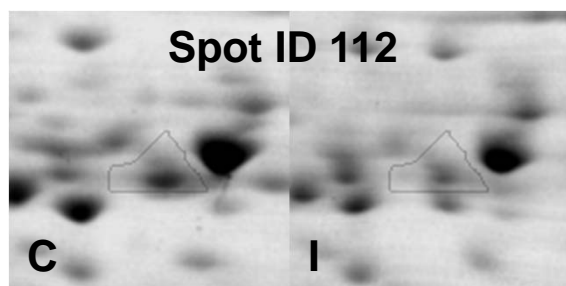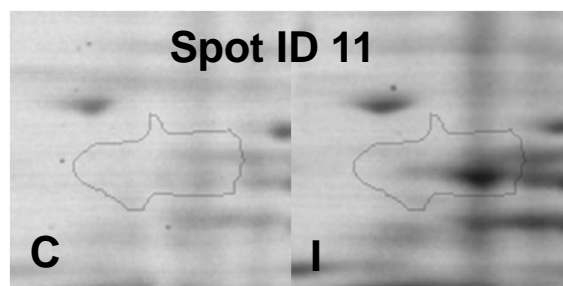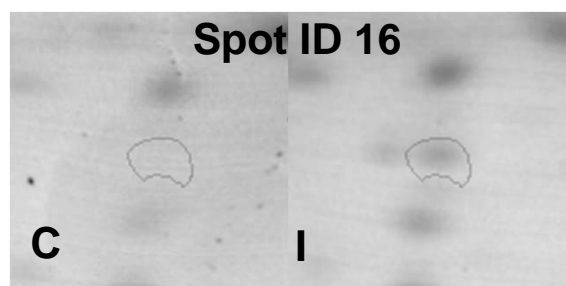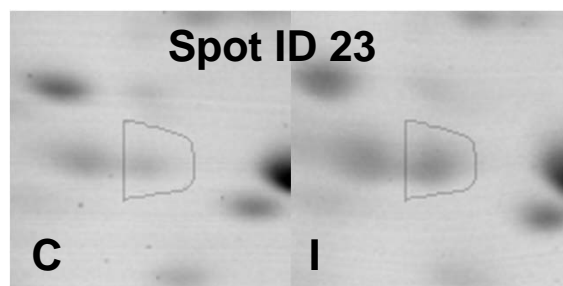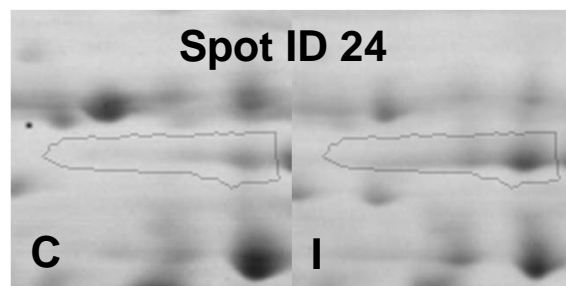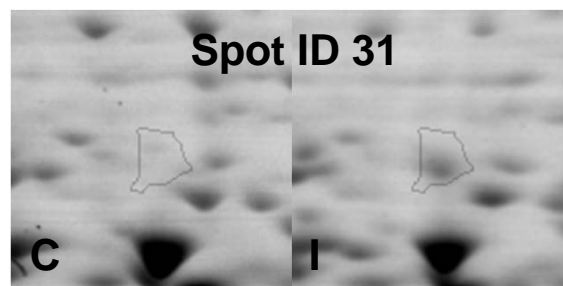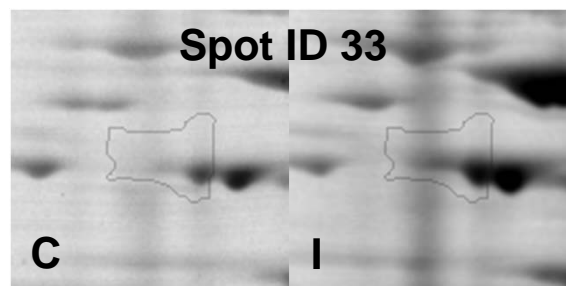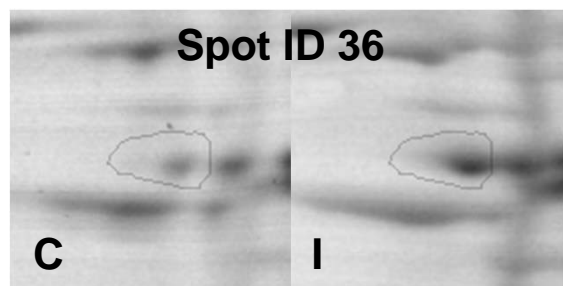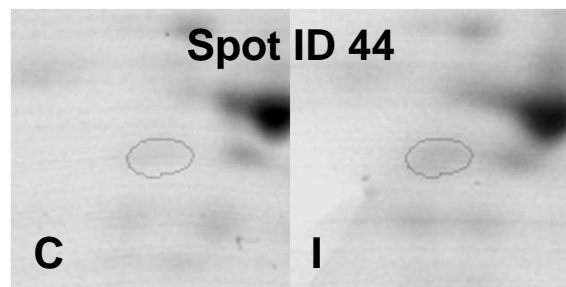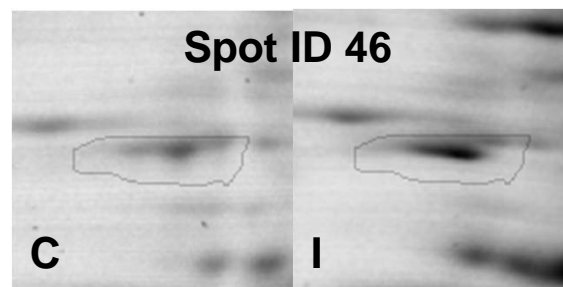

Supplementary Figure 2 continued

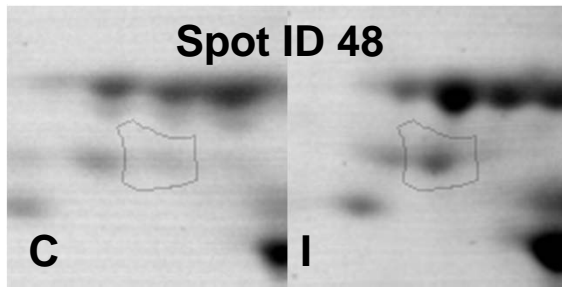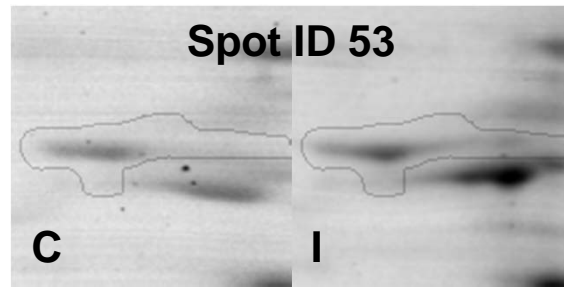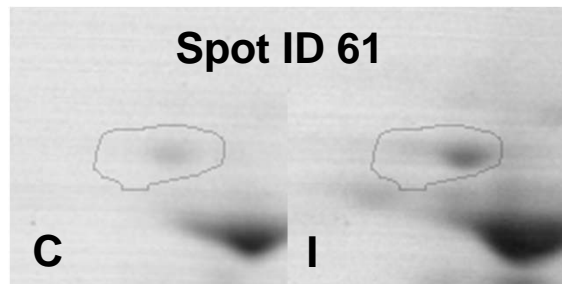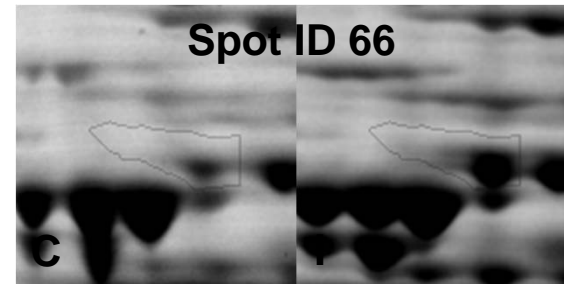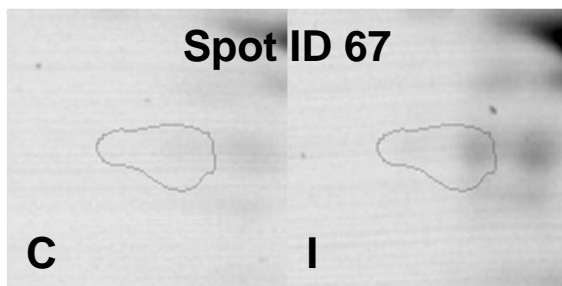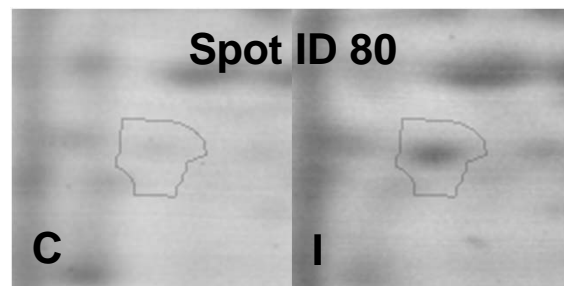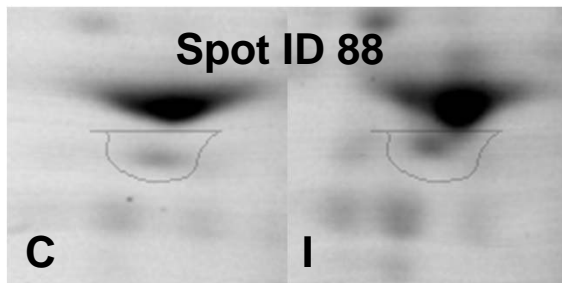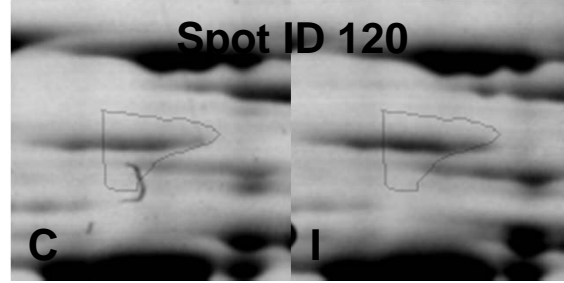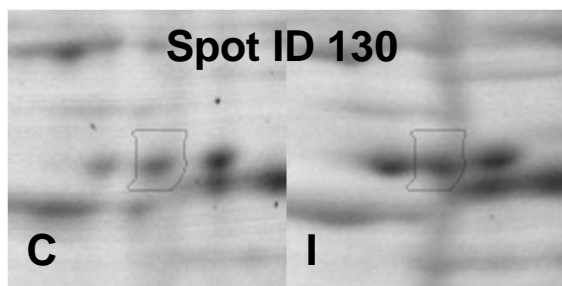

Supplement: Supplementary file 2 — Figure S2. Protein spots with significant (P < 0.05; one‐way anova) different abundance during Eurychasma dicksonii infection of the brown alga Ectocarpus siliculosus. The pictures show representative sections of control (C) and infection (I) of one 2‐DE gel. [file PCE-39-259-s002.pdf]
