# Supplementary material for: Infection of the brown alga E ctocarpus siliculosus by the oomycete E urychasma dicksonii induces oxidative stress and halogen metabolism
Source: Plant Cell Environ. 2015 Apr 23;39(2):259–71. doi: 10.1111/pce.12533 (PMC4949667; doi:10.1111/pce.12533)
Supplement: Supplementary file 3 — Table S1. Modifications made to the original protocol (Contreras et al. 2008) for protein extraction of Eurychasma dicksonii‐infected Ectocarpus siliculosus. [file PCE-39-259-s003.pdf]

Supplementary Material

Supplementary Table 1

Modifications made to the original protocol (Contreras *et al.*, 2008) for protein extraction of *Eurychasm*a *dicksonii*-infected *Ectocarpus siliculosus.*

**Original step Contreras et al. (2008)**	**Modifications (this study)**	**Comments**
Initial washing steps in MQ-H_2O and 50 mM Tris/HCl pH 8.8	omitted	based on the loss of material and the time requirement during harvest.
Joint phenolic phases (after re-extraction of the lower aqueous phase) subjected to protein precipitation	Joint phenolic phases mixed with extraction buffer (final phase extraction)	Reduction of carry-over contaminants from interphase
Protein precipitation at -20°C for 3 hr	Increased precipitation time to 15-18 hr (overnight)	Improved yield of protein precipitation
Resuspension of protein pellet in buffer containing 6 M urea	Increased concentration to 7 M urea and addition of 0.5% (v/v) Triton X-100	Improved resuspension of the protein pellet
Clean-up step following protein precipitation (2-D clean-up kit)	Clean-up step following protein quantification prior to IEF (2-D clean-up kit)	More efficient clean-up due to defined amount of protein used; easier resuspension of the protein pellet
