# Supplementary material for: Infection of the brown alga E ctocarpus siliculosus by the oomycete E urychasma dicksonii induces oxidative stress and halogen metabolism
Source: Plant Cell Environ. 2015 Apr 23;39(2):259–71. doi: 10.1111/pce.12533 (PMC4949667; doi:10.1111/pce.12533)
Supplement: Supplementary file 4 — Table S2. Oligonucleotide sequence information of Ectocarpus siliculosus genes analysed by qPCR. [file PCE-39-259-s004.pdf]

Supplementary Table 2

Oligonucleotide sequence information of *Ectocarpus siliculosus* genes analysed by qPCR

**Target gene** ***E. siliculosus******	**Primer name**	**Primer sequence 5'-3'**	**Reference**
MnSOD<br>Esi0091_0024	MnSOD for<br>MnSOD rev	CGGTGGTGTCTGAGAACTAC<br>GTGGTGGAAGATAATCGCAATG	Zambounis et al., 2013
HSP70_1<br>Esi0002_0284	HSP70_1_for<br>HSP70_1_rev	AGGCGTTGGATTGGCTGGAC<br>ACAACTCGTCGTCTCCGAAGTC	Zambounis et al., 2013
vBPO<br>Esi0009_0080	vBPO3_for<br>vBPO3_rev	GGAACTGCCCAACCTTTCGG<br>GCTCGTCACAGAAGTCGTCTCC	Zambounis et al., 2013
Pabfib<br>Esi0077_0002	PAPfib_1_F<br>PAPfib_1_R	AGACTTTGTTCTCGCTGCACC<br>ATTCGGCTGATTCGCATGT	This study
Rad23<br>Esi0017_0100	RAD23_1_F<br>RAD23_1_R	GAAGCGCTTGGTGCAGC<br>GCAGCAATTGAGCCACCTG	This study
