# Supplementary material for: Infection of the brown alga E ctocarpus siliculosus by the oomycete E urychasma dicksonii induces oxidative stress and halogen metabolism
Source: Plant Cell Environ. 2015 Apr 23;39(2):259–71. doi: 10.1111/pce.12533 (PMC4949667; doi:10.1111/pce.12533)
Supplement: Supplementary file 5 — Table S3. Protein expression in uninfected Ectocarpus siliculosus CCAP 1310/4 (‘controls’). [file PCE-39-259-s005.pdf]

Supplementary Table 3

Protein expression in uninfected *Ectocarpus siliculosus* CCAP 1310/4 (“controls”)

[a] rank Progenesis SameSpots (representative 2DE gel in Supplementary Figure 1A)
[b] *Ectocarpus siliculosus* genome
[c] based on the most significant match (Mascot)
[d] Individual ions scores > 33 indicate identity or extensive homology ($p<0.05$)

**Protein spot ID**[a]	***E. siliculosus* accession code**[b]	**Protein identification**[c]	**Number of peptides matching**	**Sequence coverage (%)**	**Theoretical MW (Da)**	**Observed / theoretical pI**	**MASCOT score**[d]
**Energy/metabolism**							
197	Esi0017_0102	Photosystem II assembly / stability factor	19	45	45078	5.04/ 5.27	586
457	Esi0002_0327	Transketolase	34	39	78568	5.32 / 5.35	587
511	Esi0311_0012	Light-harvesting complex protein	22	39	23044	4.74 / 5.2	446
531	Esi0205_0043	Aconitate hydratase	18	28	84297	5.74 / 5.37	376
536	Esi0187_0027	Triosephosphate isomerase/ Glyceraldehyde-3-phosphate dehydrogenase	30	39	62480	5.5 / 5.2	682
564	Esi0059_0038	Ferredoxin component	17	50	20521	5.62 / 6.73	331
582	Esi0022_0162	Inositol-2-Dehydrogenase	19	46	36660	5.5 / 5.22	281
597	Esi0153_0052	Cytochrome b6f complex Fe-S subunit	9	45	23846	5.37 / 6.09	144
732	Esi0240_0024	Glyceraldehyde-3-phosphate dehydrogenase	52	59	40987	5.78/ 5.92	735

Protein spot ID[a]	*E. siliculosus* accession code[b]	Protein identification[c]	Number of peptides matching	Sequence coverage (%)	Theoretical MW (Da)	Observed / theoretical pI	MASCOT score[d]
736	Esi0112_0005	Ferredoxin-NADP oxidoreductase	14	35	46059	5.87 / 8.71	140
714	Esi0009_0009	Peroxidase (Haem / cytochrome c)	22	14	38366	6.465 / 7.66	582
769	Esi0155_0030	Manganese-stabilising protein	61	62	34241	5.44 / 5.3	1466
786	Esi0112_0005	Ferredoxin-NADP oxidoreductase	30	35	46059	5.93/ 8.71	514
803	Esi0038_0073	FeS assembly protein	8	18	56405	5.44 / 5.18	129
848	Esi0011_0185	Pyruvate carboxylase	8	9	125080	4.97 / 5.71	144
852	Esi0000_0409	ATP synthase y chain	23	45	40739	5.78 / 5.92	882
**Defence and cell rescue**							
724	Esi0091_0024	Manganese superoxide dismutase	4	12	32055	5.78/ 6.34	166
**Protein folding**							
723	Esi0279_0030	Heat shock protein 20	13	63	21775	5.24 / 5.18	286
543	Esi0155_0059	Protein disulfide isomerase	2	14	38436	6.61 / 6.56	92
745	Esi0274_0029	Phosphoadenylyl-sulfate reductase (Thioredoxin)	20	41	35608	5.32 / 5.29	530
**Cellular organization**							
297	Esi0126_0019	Flagellar-associated protein	32	52	40267	5.40 / 8.45	618
305	Esi0203_0038	Actin	31	62	42106	5.65 / 5.3	669
**Unknown, hypothetical proteins**							
166	Esi0159_0027	Putative RNA binding protein	11	15	65826	6.27 / 5.99	146
432	Esi0035_0130	PAP/fibrillin domain	11	39	38778	4.69 / 4.98	481

Protein spot ID[a]	*E. siliculosus* accession code[b]	Protein identification[c]	Number of peptides matching	Sequence coverage (%)	Theoretical MW (Da)	Observed / theoretical pI	MASCOT score[d]
		containing protein					
592	Esi0403_0006	Conserved hypothetical protein	1	2	31175	6.07 / 7.66	35
741	Esi0208_0011	Putative RNA binding protein	8	19	47251	5.32 / 5.54	367
